# Characterization of Metal-Bound Benzimidazole Derivatives, Effects on Tumor Cells of Lung Cancer

**DOI:** 10.3390/ma14112958

**Published:** 2021-05-30

**Authors:** Anita Raducka, Agnieszka Czylkowska, Katarzyna Gobis, Kamila Czarnecka, Paweł Szymański, Marcin Świątkowski

**Affiliations:** 1 Institute of General and Ecological Chemistry, Faculty of Chemistry, Lodz University of Technology, Zeromskiego 116, 90-924 Lodz, Poland; marcin.swiatkowski@p.lodz.pl; 2 Department of Organic Chemistry, Faculty of Pharmacy, Medical University of Gdansk, Gen. Hallera 107, 80-416 Gdansk, Poland; katarzyna.gobis@gumed.edu.pl; 3 Department of Pharmaceutical Chemistry, Drug Analyses and Radiopharmacy, Faculty of Pharmacy, Medical University of Lodz, Muszynskiego 1, 90-151 Lodz, Poland; kamila.czarnecka@umed.lodz.pl (K.C.); pawel.szymanski@umed.lodz.pl (P.S.); 4 Department of Radiobiology and Radiation Protection, Military Institute of Hygiene and Epidemiology, 4 Kozielska St., 01-163 Warsaw, Poland

**Keywords:** lung cancer, copper (II) coordination compounds, benzimidazole derivatives, thermogravimetric analysis

## Abstract

Four new ligands and four new copper (II) coordination compounds were prepared and characterized by chemical, elemental analysis, cytotoxicity, and FTIR spectroscopy (Fourier transform infrared spectroscopy). The nature of metal–ligand coordination was investigated. The thermal properties of complexes in the solid state were studied using TG-MS techniques (thermogravimetric analysis coupled with mass spectrometry) under dynamic flowing air atmosphere to analyze the principal volatile thermal decomposition and fragmentation products that evolved during thermolysis. The intermediate and final solid thermolysis products were also determined. The MTT (3-(4,5-dimethylthiazol-2-yl)-2,5-diphenyltetrazoliumbromide) assay was used to evaluate active metabolic cells as an IC_50_ (half maximal inhibitory concentration). The relationship between antitumor activity and the position of nitrogen atoms in the organic ligand has been shown.

## 1. Introduction

The year 2020 focused the scientific community on the COVID-19 pandemic [1,2,3]. The pandemic has accelerated the search for new drugs, not forgetting one of the most deadly cancers—lung cancer. The main goal was to inhibit the number of diseases and the spread of the virus while looking for drugs for existing diseases [4,5,6,7]. Scientists around the world have been searching and are still looking for pharmaceutical compounds with potential antifungal, antibacterial, anticancer, and antiviral properties, with particular emphasis on coronavirus (2019-nCoV), which emerged in Wuhan, China [8,9,10]. The higher risk of serious complications and death from SARS-CoV-2 coronavirus infection particularly affects patients with lung cancers, leukemia, lymphomas, multiple myeloma, and gastrointestinal cancers [11,12,13,14,15,16,17,18,19,20,21,22,23,24]. People with cancer are more likely to experience severe COVID-19 and dangerous complications. However, research reports that certain types of cancer increase the risk of dying from a virus [25,26]. Additionally, as a result of the epidemic, another major problem has arisen in the treatment of cancer. Treatments are delayed, pain medications are lacking, and some cancer-related clinical trials are stopped. The ability to undergo surgeries and treatments has also been drastically reduced [27,28,29]. It seems justified to continue searching for potential drugs for diseases that kill most people. The modern therapeutic agents containing metal complexes comprise a fundamental class of drugs for treating tumors and other disease. Many metal-containing drugs based on gold, ruthenium, gallium, titanium, and iron are in preclinical and clinical trials. It is commonly known that antitumor drugs based on endogenous metal such as cooper are less toxic as compared for example with platinum analogues. Coordination compounds with cooper are promising antitumor therapeutic agents. Its various mechanisms such as inhibition of proteasome activity, telomerase activity, reactive oxygen species, DNA degradation, and DNA intercalation make them promising in anticancer therapy [30]. Lung cancer is the most dangerous and common cancer. It ranks first in terms of the number of cases and mortality. One of the hallmarks of a malignant lung cancer is its ability to metastasize, which increases mortality among patients [31,32,33]. Benzimidazoles and their derivatives are widely used all over the world due to their biological activity [34,35,36]. Particularly important are those derivatives that show potential activity and the possibility of using them in anticancer therapy [37,38,39,40]. Complexes of imidazole and benzimidazole ligands have received a lot of attention. This work describes a new series of derivatives benzimidazoles that were rationally designed and synthesized by combining different anticancer fragments with copper, which is used as an antibacterial, antifungal, or antiviral agent. As a result of the synthesis, four coordination compounds were obtained. Their stoichiometric composition, metal–organic ligand bond nature, thermal decomposition, and volatile compounds emitted during dry air thermolysis as well as FTIR spectra and anticancer properties are also described here. The compounds were also tested for percent viability of cells by MTT assay.

## 2. Materials and Methods

### 2.1. Materials and Analysis

Substrates used for ligand synthesis as well as CuCl_2_·2H_2_O were purchased from Sigma Aldrich (Warszawa, Poland).

For the cytotoxicity assays, a human lung adenocarcinoma from the European Collection of Cell Cultures (ECACC, Salisburg, UK), A549, were used. A549 cells were cultured in Dulbecco’s Modified Eagle’s Medium (PAN-Biotech, Aidenbach, Germany) and 10% fetal bovine serum (FBS) (Sigma Aldrich), 2 mM glutamine (Sigma Aldrich) and 100 units/mL penicillin and 100 mg/mL streptomycin (Sigma Aldrich) in humidified atmosphere with 5% of CO_2_, at 37 °C.

### 2.2. Methods and Instruments

Samples of complexes (about 20 mg) were digested in a mixture of concentrated 36% HCl (1 mL) and 65% HNO_3_ (6 mL). The contents of Cu (II) in solid complexes were determined by the F-AAS spectrometer (Analityk Jena, contraAA 300, Jena, Germany) with a continuum source of light and using air/acetylene flame (Analityk Jena, contraAA 300). Absorbances were measured at analytical spectral lines 324.7 nm for Cu (II). The limit of quantification was 0.04 mg/L for Cu (II). Solid samples were decomposed using the Anton Paar Multiwave 3000 (Graz, Austria) closed system instrument. Mineralization was carried out for 45 min at 240 °C under pressure 60 bar. The contents of carbon, hydrogen, and nitrogen were determined by a Vario micro company Elementar Analysensysteme GmbH, (Langenselbold, Germany). FTIR spectra were recorded with an IR Tracer-100 Schimadzu Spectrometer (4000–600 cm^−1^ with an accuracy of recording of 1 cm^−1^, Kyoto, Japan) using KBr pellets. The thermal analyses were carried out with a Netzsch STA 449 F1 Jupiter thermoanalyzer (Netzsch-Geratebau GmbH, Selb, Germany) coupled with Netzsch Aeolos Quadro QMS 403 mass spectrometer (Netzsch-Geratebau GmbH, Selb, Germany). Samples were heated in corundum crucibles up to 1000 °C, with 10 °C/min heating rate, in the atmosphere of synthetic air (20% O_2_, 80% N_2_).

For the final assay, cells were seeded into standard 96-well plates at a density of 10^4^ cells per well. Next, they were cultured for 24 h at 37 °C and 5% CO_2_. After 24 h, the medium was removed, and cells were exposed to the 100 µL of the compounds and another 24 h of incubation. In the next step, the medium was removed, and 50 µL of the MTT solution was added to each well and for additional 2 h at 37 °C. Then, the solution from cells was carefully removed, and 100 µL of DMSO was added. After incubation (10 min), the Sorensen Buffer was added to each well (5 µL). The final absorbance was measured by using microplate reader (Synergy H1, BioTek, Winooski, VT, USA). Results were expressed as a percentage of the control values (blank). To the assay, MTT (Sigma Aldrich) was dissolved at a concentration of 0.5 mg/mL in phosphate-buffered saline (PBS) (PAN-Biotech). Sorensen’s glycine buffer is made of 0.1 M glycine and 0.1 M NaCl, at pH 10.5. All analyzed compounds were dissolved in DMSO in order to receive stock solutions and prepared dissolutions (final DMSO concentration was below 0.2%). In the blank control nothing but only culture medium was used. Pure DMSO was used as a control [41].

### 2.3. Statistical Analysis

All values were prepared with ± standard deviation (SD) of the obtained experiments. One-way ANOVA was performed using Stat-Soft STATISTICA v.13.1 software (TIBCO, Palo Alto, CA, USA). We used post hoc analysis too.

## 3. Results and Discussion

### 3.1. Synthesis

#### 3.1.1. Ligand Synthesis

**Scheme 1:**
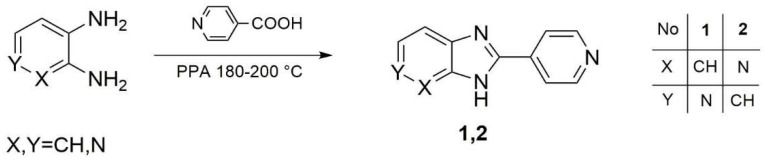
Synthesis of ligand 1 (**L1**) and 2 (**L2**).

Ligands 1 and 2 were synthesized by the condensation and cyclization of 2,3-diminopyridine or 3,4-diaminopyridine with isonicotinic acid in PPA (Scheme 1). The general procedure for the synthesis of imidazopyridines 1,2: 0.54 g (5 mmol) of appropriate diamine, 0.98 g (8 mmol) of isonicotinic acid, and 20 g of PPA was stirred with heating at 180–200 °C for 5 h. Then, the mixture was poured onto 100 g of ice, alkalized with solid NaHCO_3_, filtered, and washed with water.

##### 2-(Pyridin-4-yl)-3*H*-imidazo[4,5-*c*]pyridine (**L1**)

The cured product was crystallized from EtOH/water mixture (1:1); yield: 0.55 g (61%); mp. 282–283 °C; IR: 3392, 1611, 1451, 1428, 1329, 1316, 1010, 815, 706, 633 cm^−1^; ^1^H NMR: (500 MHz, DMSO-d6): 7.68 (d, 1H, 3-Py, J = 5 Hz), 8.15 (d, 2H, 4-Py, J = 6 Hz), 8.36 (d, 1H, 3-Py, J = 5.5 Hz), 8.80 (d, 2H, 4-Py, J = 6 Hz), 9.04 (s, 1H, 3-Py), 13.72 (brs, 1H, NH with D_2_O exchangeable) ppm.

##### 2-(Pyridin-4-yl)-3*H*-imidazo[4,5-*b*]pyridine (**L2**)

The cured product was crystallized from dioxane; yield: 0.6 g (61%); mp. 308–309 °C; IR: 3423, 3085, 1605, 1435, 1407, 1375, 1265, 828, 766, 608, 607 cm^−1^; ^1^H NMR: 7.30 (dd, 1H, 2-Py, J_1_ = 5 Hz, J_2_ = 3 Hz), 8.14–8.15 (m, 3*H*, 2H 4-Py and 1H 2-Py), 8.42 (m, 1H, 2-Py), 8.79 (d, 2H, 4-Py, J = 6 Hz), 13.90 (brs, NH with D_2_O exchangeable) ppm.

**Scheme 2:**
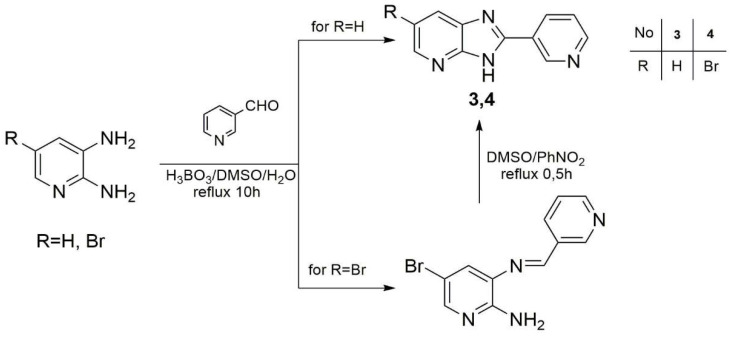
Synthesis of ligand 3 (**L3**) and 4 (**L4**).

In contrast, ligands 3 and 4 were obtained by reacting 2,3-diaminopyridine or 5-bromo-2,3-diaminopyridine with 3-pyridinecarboxaldehyde in the presence of boric acid in a mixture of water and DMSO (Scheme 2). In the case of the bromo derivative (4), under these conditions, the reaction only proceeded to obtain an imine, which was then cyclized in a mixture of nitrobenzene and DMSO. The structures of the compounds obtained were confirmed by infrared spectroscopy and ^1^H nuclear magnetic resonance.

##### 2-(Pyridin-3-yl)-3*H*-imidazo[4,5-*b*]pyridine (**L3**)

To the solution of 0.3 g (5 mmol) of boric acid in 3 mL of water, 0.47 mL (0.54 g, 5 mmol) of 3-pyridinecarboxaldehyde and 0.54 g (5 mmol) of 2,3-diaminopyridine in 3 mL of DMSO were added. The mixture was refluxed for 10 h. Then, 20 g of ice was added, precipitate was filtered off and washed with water. The crude product was recrystallized from EtOH/H_2_O (1:1) mixture; yield: 0.21 g (21%); mp. 296–297 °C. IR: 3045, 3005, 2922, 2853, 2788, 2681, 1573, 1442, 1413, 1321, 1282, 946, 772, 713 cm^−1^; ^1^H NMR (200 MHz, CDCl_3_): 7.39 (q, 1H, 2-Py, J = 3 Hz), 7.57 (dd, 1H, 3-Py, J_1_ = 5 Hz, J_2_ = 3 Hz), 8.22 (d, 1H, 3-Py, J = 7 Hz), 8.54 (d, 1H, 2-Py, J = 4 Hz), 8.62 (d, 1H, 3-Py, J = 8 Hz), 8.82 (d, 1H, 2-Py, J = 4 Hz), 9.54 (d, 1H, 3-Py, J = 1 Hz) ppm; NH—invisible in spectrum.

##### 6-Bromo-2-(pyridin-3-yl)-3*H*-imidazo[4,5-*b*]pyridine (**L4**)

To the solution of 0.3 g (5 mmol) of boric acid in 3 mL of water 0.47 mL (0.54 g, 5 mmol) of 3-pyridinecarboxaldehyde and 0.94 g (5 mmol) of 5-bromo-2,3-diaminopyridine in 3 mL of DMSO were added. The mixture was refluxed for 10 h. Then, 20 g of ice was added, and yellow precipitate (1.1 g, 83%) was filtered off and washed with water. The crude imine (0.57 g, 2 mmol), 2 mL of nitrobenzene, and 2 mL of DMSO were refluxed for 0.5 h. Then, 20 g of ice was added, precipitate was filtered off, and 20 mL of diethyl ether was added to the filtrate. Another portion of precipitate was filtered off. The crude product was recrystallized from dioxane/petroleum ether (1:1) mixture; yield: 0.3 g (55%); mp. > 220 °C; IR: 2920, 2852, 1449, 1390, 1277, 947, 933, 703 cm^−1^; ^1^H NMR (200 MHz, DMSO-d6): 7.61–7.67 (m, 1H, 3-Py), 8.37–8.77 (m, 4H, 2H 3-Py and 2H 2-Py), 9.40 (s, 1H, 3-Py), 13.84–13.94 (brs, 1H, NH with D_2_O exchangeable) ppm.

#### 3.1.2. Complex Synthesis

**Scheme 3:**
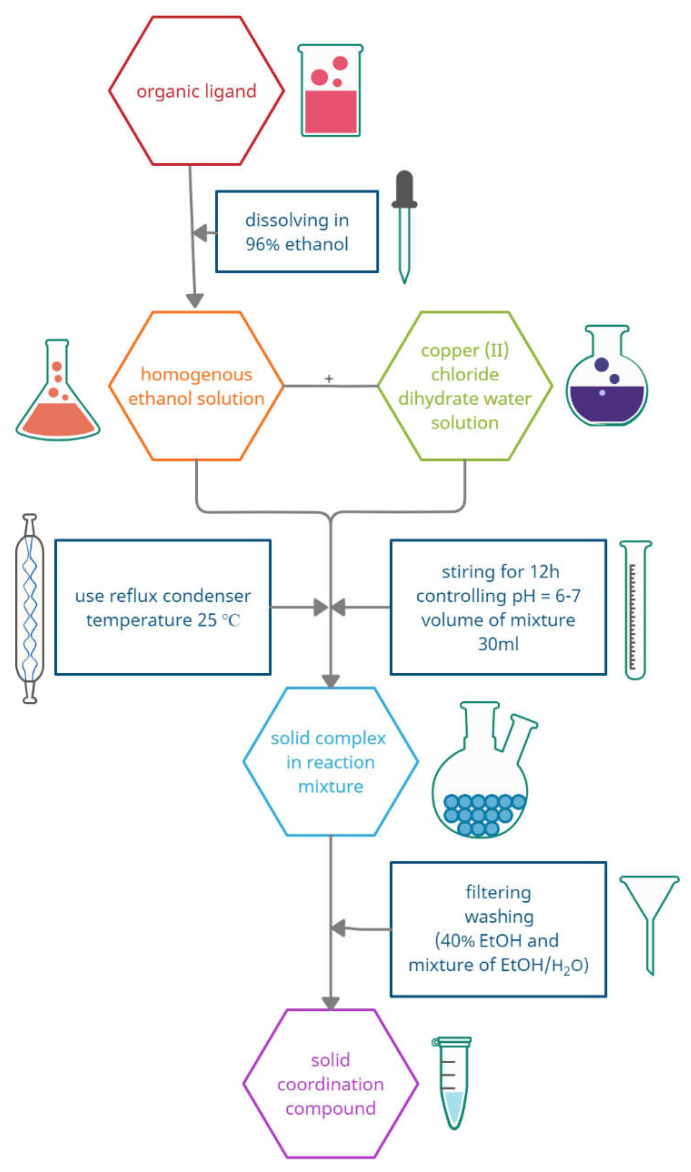
Synthesis of solid copper (II) coordination compounds.

Dissolving the amount of organic ligands assumed for synthesis of copper (II) complexes required the use of a reflux condenser and a magnetic stirrer for about 12 h. The reactions were carried out at room temperature (25 °C). The mixed-ligand complexes were prepared by mixing 0.25 mmol of the appropriate ligand in 96% *v*/*v* ethanol with freshly obtained ethanol solutions of 0.25 mmol copper (II) chlorides dihydrate (Scheme 3). The total volume of the reaction mixtures was 30 mL. After obtaining homogenous solutions, the reaction mixtures were stirred for 12 h until precipitation occurred. All steps were performed at a controlled pH (6–7) and room temperature. The obtained complexes were washed with 40% EtOH and then with a mixture EtOH and H_2_O (*v*/*v* = 1/1). The products were air-dried at room temperature. The newly obtained complexes were characterized by elemental C/H/N analysis and Cu (II) content determination, FTIR spectra, and TG-MS study.

**Cu(L1)Cl_2_∙H_2_O (C_11_H_10_N_4_OCuCl_2_)** (348.6795 g/mol), yield (46%), anal. calculated (%): Cu, 18.22; C, 37.89; H, 2.89; N, 16.34. Found (%): Cu, 18.75; C, 37.12; H, 2.86; N, 16.46.

**Cu(L2)Cl_2_∙H_2_O (C_11_H_10_N_4_OCuCl_2_)** (348.6795 g/mol), yield (41%), anal. calculated (%): Cu, 18.22; C, 37.89; H, 2.89; N, 16.34. Found (%): Cu, 17.92; C, 37.34; H, 2.91; N, 16.39.

**Cu(L3)Cl_2_ (C_11_H_7_N_4_CuCl_2_)** (330.6642 g/mol), yield (39%), anal. calculated (%): Cu, 19.22; C, 39.95; H, 2.13; N, 17.02. Found (%): Cu, 19.66; C, 39.81; H, 2.10; N, 16.81.

**Cu(L4)Cl_2_∙H_2_O (C_11_H_9_N_4_OCuBrCl_2_)** (428.5835 g/mol), yield (44%), anal. calculated (%): Cu, 14.82; C, 30.82; H, 2.12; N, 13.13. Found (%): Cu, 14.62; C, 30.69; H, 2.15; N, 13.54.

### 3.2. MTT Cytotoxicity Assay

The MTT assay was used to evaluate (by thiazolyl blue tetrazolium bromide) active metabolic cells as an IC_50_ (the concentration of compounds required for 50% inhibition of cell growth) were calculated by concentration trend lines curve using a Microsoft Office Excel (Microsoft, Redmond, Washington, DC, USA). This test is based on the determination of value cell viability by measuring the metabolic activity. The activity is measure in mitochondria. The mitochondria reduce the water-soluble MTT (3-(4,5-dimethylthiazol-2-yl)-2,5diphenyltetrazoliumbromide) to blue-violet insoluble formazan, which is determined by spectrophotometry. Results are as a number of viability cells. It is proportional to the color intensity determined by photometric measurements. The standard analytical wavelength for this complex is 570 nm in a mixture of dimethylsulfoxide (DMSO) (Serva) and Sorensen’s glycine buffer. The experiments were done in triplicate [42]. The concentration of analyzed compounds was used to determine IC_50_ values. The typical chemotherapeutic agent against lung cancer used in clinical therapy, etoposide, was used as positive controls. All tested derivatives showed cytotoxicity activity (Table 1) with the IC_50_ values in the range from 264.32 to 608.70 μM against cancer cell lines. Controls compound possessed middle cytotoxicity activity compared with the tested derivatives—etoposide IC_50_ 451.47 μM. For the A549 cell line, IC_50_ for new derivatives (complex 2—319.33 +/− 31.08 μM; complex 3—342.03 +/− 17.69 μM, complex 4—264.32 +/− 36.57 μM) values were lower than for etoposide, which indicated that colorectal adenocarcinoma cells were more sensitive to analyzed compounds than derivative complex 1—608.7 +/− 65.17 μM. Complex 4—IC_50_ 264.32 +/− 36.57 μM against A549 was the most cytotoxic compound against colorectal adenocarcinoma cells. The obtained results are statistically significant. Against lung cancer cells, the structure–activity relationship showed that the position of nitrogen atom in imidazo[4,5-*c*]pyridine rings is very important. Additionally, the position of the nitrogen atom in the phenolic ring is of great importance. However, it is the bromine atom at the 6-position of the imidazo[4,5-*c*]pyridine rings that causes the significantly increased cytotoxic activity.

### 3.3. FTIR Spectra

**Figure 1:**
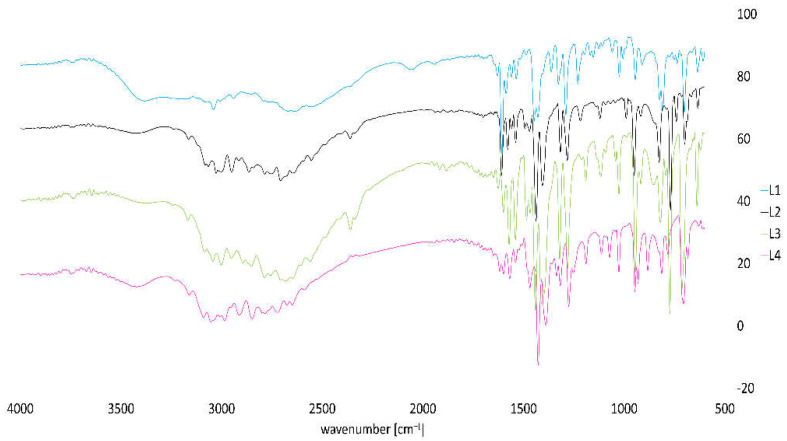
FTIR spectrum of free organic ligands.

**Figure 2:**
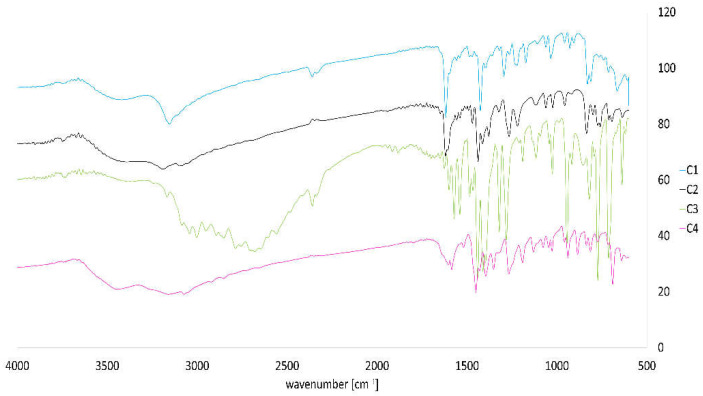
FTIR spectrum of copper (II) coordination compounds.

Figure 1 and Figure 2 present the FTIR spectrum of free organic ligands and their copper (II) complexes. The spectrum of the ligands shows strong bands between 3200 and 2650 cm^−1^, due to *ν*(NH), being related to the presence of the imidazole ring. In the spectra of complexes C1, C2, and C4, they are moved to higher wavenumbers (3190–3066 cm^−1^), indicating the presence of the NH group, which does not take part in the bonding. The exception is compound 3, for which they do not change position in comparison to the free ligand. In the absorption region of N-donor ligands appear modes of *ν*(C=N) and *ν*(C=C) in the ranges 1627−1600 and 1483−1390 cm^−1^, respectively. They are moved to higher and lower frequencies as a result of coordination between copper (II) ions and organic ligands. In the case of complex 3, the *ν*(C=C) modes are in a similar range as for free ligand and prove different coordination between copper (II) and organic ligand (**L3**). The CH stretching vibrations overlap with *ν*(NH) bands in the spectra of all compounds. For uncoordinated ligand, there are also modes of β(CH) and γ(CH) between 1294–1173 cm^−1^ and 850−690 cm^−1^, respectively. In the complexes, they appear in similar ranges. The occurrence of OH stretching band in the spectrum of complexes 1, 2, and 4 at about 3440 cm^−1^ indicates the presence of water molecules.

Thus, it is clear from the FTIR data that N-donors act as monodentate ligands in complexes C1, C2, and C4. In the case of copper (II) compound C3, benzimidazole coordinates through the nitrogen atoms of the pyridine rings and weak interaction between metal (II) and hydrogen from the imidazole ring.

### 3.4. Thermal Study

**Figure 3:**
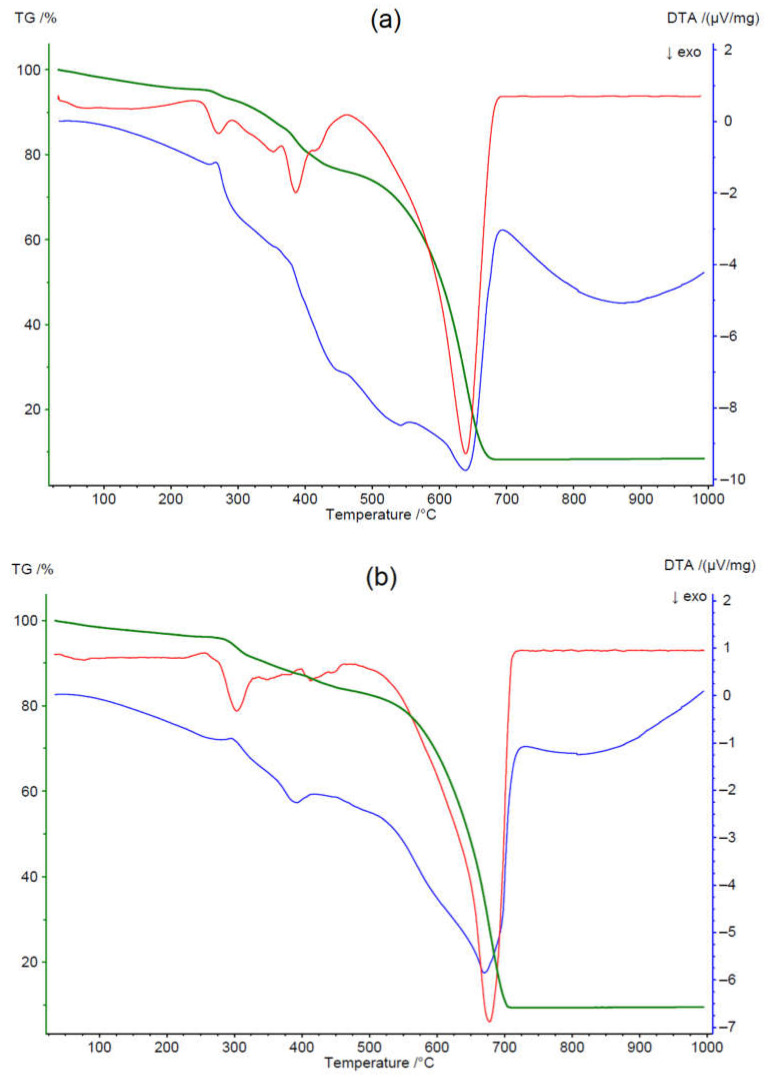

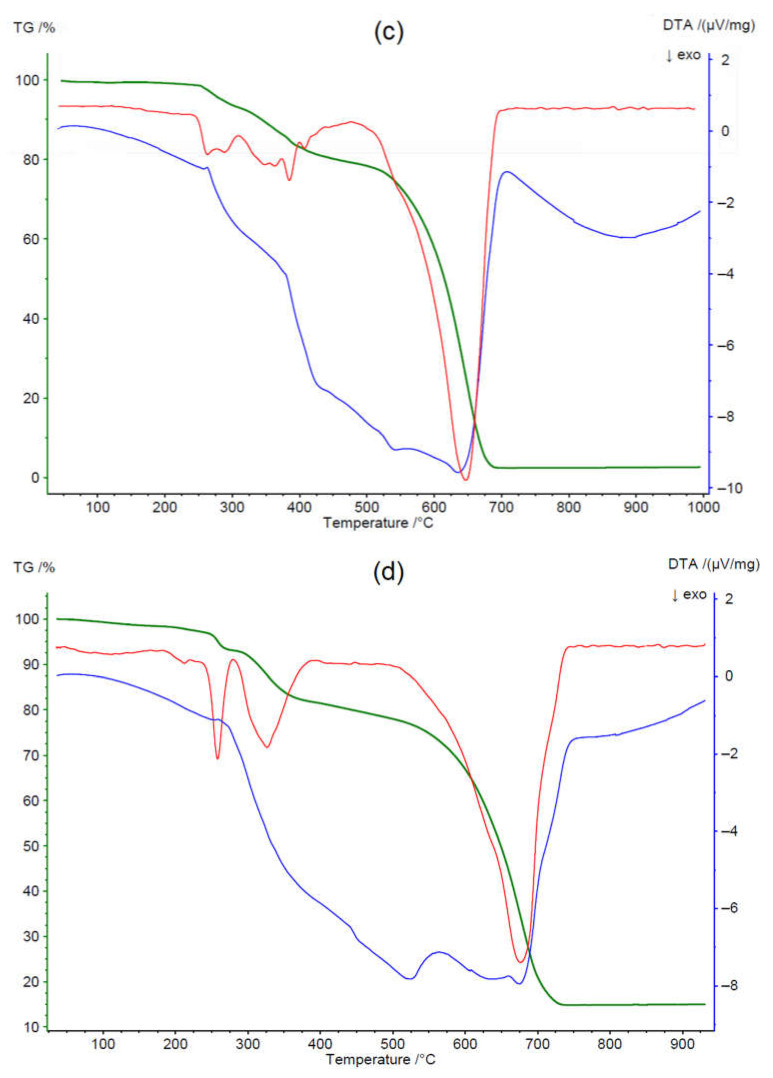
TG (green), DTA (blue), and DTG (red) curves for copper (II) complexes C1 (**a**), C2 (**b**), C3 (**c**), and C4 (**d**).

**Figure 4:**
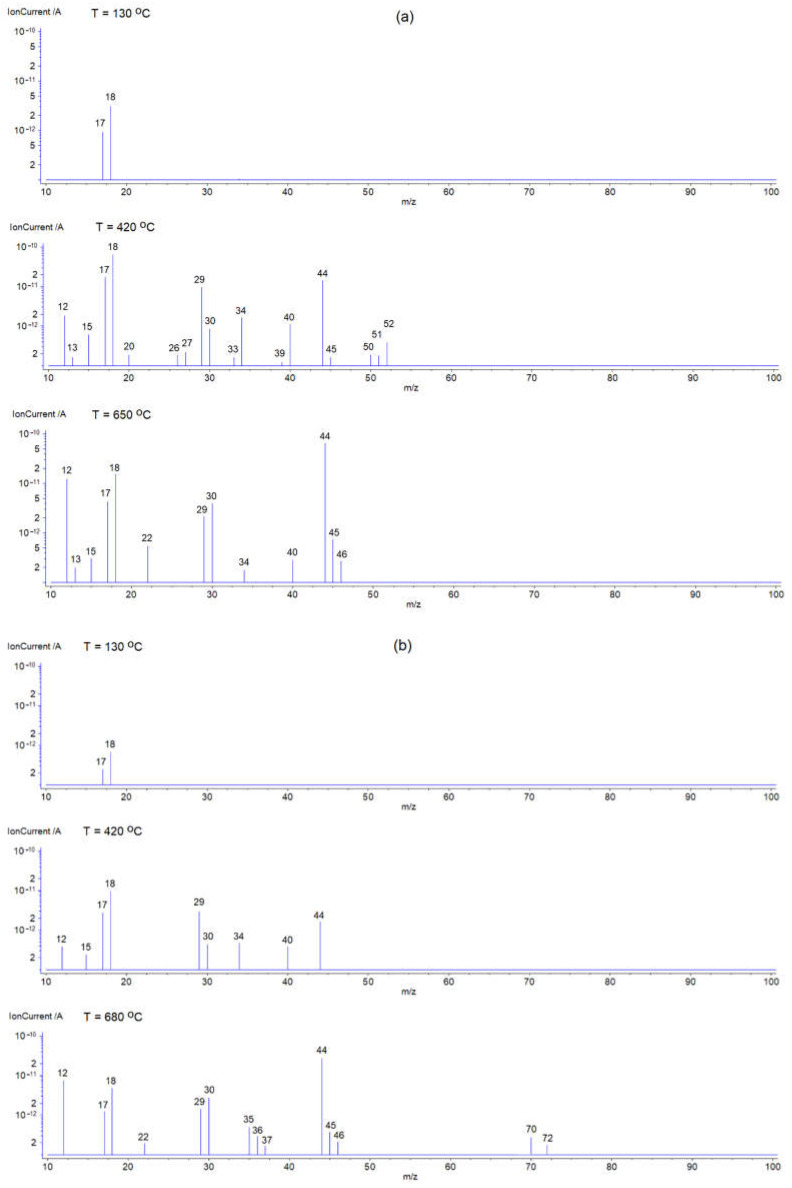

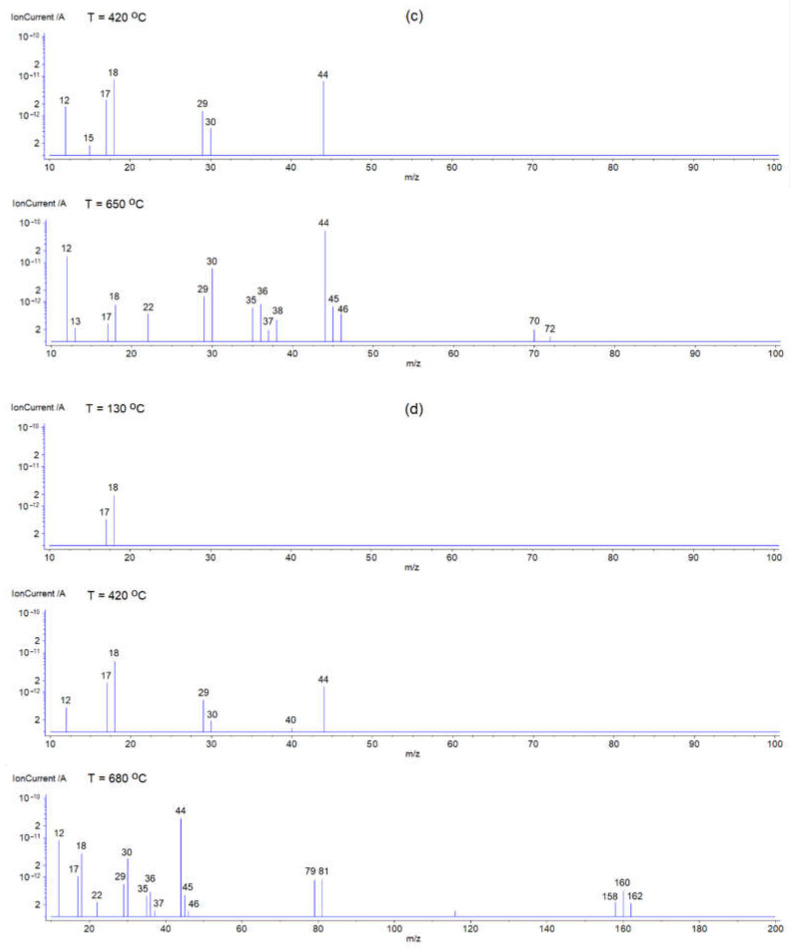
Mass spectra of volatile products from the thermal decomposition of complexes C1 (**a**), C2 (**b**), C3 (**c**), and C4 (**d**). The elaboration of the mass spectra involved subtracting the background spectrum and the application of the automatic software correction for the carrier gas.

Studied compounds decompose thermally according to the same pathway (Figure 3). The first stage for compounds containing water is dehydration (Figure 4). It is a continuous process without steep mass loss. In the second stage (first for complex 3), the non-conjugated pyridine ring of the organic ligand decomposes. The fragmentation ions characteristic for pyridine such as C_4_H_4_^+^ (*m/z*: 50, 51, 52) and HCN^+^ (*m/z*: 26, 27) [43] were registered in the mass spectrum from this stage for complex 1 (Figure 4a). In addition, the typical volatile products for the oxidation of heterocyclic organic species are present (C^+^, OH^+^, H_2_O^+^, NO^+^, CO_2_^+^). The rest of the organic ligand decomposes in the third stage (second for complex 3). It is especially visible for complex 4, which contains bromide substituent. The signals of Br^+^ (*m/z*: 79, 81) and Br_2_^+^ (*m/z*: 158, 160, 162) were detected in the mass spectrum from this stage (Figure 4d). Chloride anions are removed simultaneously with organic ligand oxidation in the last stage, which is confirmed by the presence of Cl^+^ (*m/z*: 35, 36, 37) and Cl_2_^+^ (*m/z*: 70, 72) volatile products (Figure 4). The final product is CuO in all cases. The residual mass is significantly lower than the expected value for all studied compounds (theoretical residual mass is around 20%). It is a consequence of the formation of Cu_x_Cl_x_ volatile species [44]. The release of various amounts of Cu_x_Cl_x_ is the reason why those residual masses are not comparable between studied compounds.

## 4. Conclusions

Four new organic ligands and their coordination compounds with copper (II) were obtained. On the basis of elemental analysis and infrared studies, the stoichiometric formulae of the synthesized complexes were determined.

The types of metal–organic ligand bonding were determined. In complexes C1, C2, and C4, N-donors act as monodentate ligands. In the case of C3, benzimidazole coordinates through the nitrogen atoms of the pyridine rings with a weak interaction between metal (II) and hydrogen from the imidazole ring. Thermal studies provided information on their thermal stability and showed data of volatile decomposition and fragmentation products. All tested derivatives showed cytotoxicity activity. Our findings provide useful information for the further development of 6-bromo-2-(pyridin-3-yl)-3*H*-imidazo[4,5-*b*]pyridine (C4) copper (II) complexes derivatives as promising lead compounds in research as potential anti human lung adenocarcinoma drug candidates. In addition, the MTT assay was used to investigate the cytotoxicity of the new compounds. Compound C4 showed strong activity against the cell line (A549 cells).

A large number of people suffering from lung diseases, including lung cancer, is the main reason for ongoing research into drugs for these kinds of diseases. It is also important to point out that these numbers are growing very rapidly. It is a crucial task to investigate new coordination compounds for their biological activity, especially when many drugs that are currently in use become less effective against certain types of bacteria or viruses, which is a well-known phenomenon.

## Figures and Tables

**Table 1:** Cytotoxicity activity with the IC_50_ values. Statistical significance was assessed using one-way ANOVA analysis was performed.

Complex	Cytotoxicity Activity [μM]
1	608.7 +/− 65.17 *
2	319.33 +/− 31.08 *
3	342.03 +/− 17.69 *
4	264.32 +/− 36.57 *
etoposide	451.47 +/− 18.27

* *p* < 0.01 was considered as significantly different between cytotoxicity of etoposide and tested compounds.

## Data Availability

Not applicable.
